# Diagnostic Value of Software-Based Image Fusion of Computed Tomography and F18-FDG PET Scans in Patients with Malignant Lymphoma

**DOI:** 10.1100/2012/821694

**Published:** 2012-05-02

**Authors:** B. Henninger, D. Putzer, D. Kendler, C. Uprimny, I. Virgolini, E. Gunsilius, R. Bale

**Affiliations:** ^1^Department of Radiology, Medical University Innsbruck, 6020 Innsbruck, Austria; ^2^Department of Nuclear Medicine, Medical University Innsbruck, 6020 Innsbruck, Austria; ^3^Division of Hematology and Oncology, Department of Internal Medicine, Medical University Innsbruck, 6020 Innsbruck, Austria

## Abstract

*Aim*. The purpose of this study was to evaluate the accuracy of 2-deoxy-2-[fluorine-18]fluoro-D-glucose (FDG) positron emission tomography (PET), computed tomography (CT), and software-based image fusion of both modalities in the imaging of non-Hodgkin's lymphoma (NHL) and Hodgkin's disease (HD). *Methods*. 77 patients with NHL (*n* = 58) or HD (*n* = 19) underwent a FDG PET scan, a contrast-enhanced CT, and a subsequent digital image fusion during initial staging or followup. 109 examinations of each modality were evaluated and compared to each other. Conventional staging procedures, other imaging techniques, laboratory screening, and follow-up data constituted the reference standard for comparison with image fusion. Sensitivity and specificity were calculated for CT and PET separately. *Results*. Sensitivity and specificity for detecting malignant lymphoma were 90% and 76% for CT and 94% and 91% for PET, respectively. A lymph node region-based analysis (comprising 14 defined anatomical regions) revealed a sensitivity of 81% and a specificity of 97% for CT and 96% and 99% for FDG PET, respectively. Only three of 109 image fusion findings needed further evaluation (false positive). *Conclusion*. Digital fusion of PET and CT improves the accuracy of staging, restaging, and therapy monitoring in patients with malignant lymphoma and may reduce the need for invasive diagnostic procedures.

## 1. Introduction

The prognosis of Hodgkin's disease (HD) and non-Hodgkin's lymphoma (NHL) has significantly improved in recent years. This is a result not only of new therapeutic concepts, for example, the implementation of Rituximab, but also of accurate staging procedures and the early evaluation of response during treatment. For staging and monitoring, computed tomography (CT) is commonly used, but its information is limited to anatomical criteria such as the size of lymph nodes. Thus, for small lymph nodes malignancy may be overlooked. Nevertheless, CT is still the most commonly available technique for identification of nodal involvement, as well as for staging and restaging of malignant lymphoma [1].

Positron emission tomography (PET) with 2-deoxy-2-[fluorine-18] fluoro-D-glucose (FDG) is an established imaging technique for malignant tumors [2–4] including malignant lymphoma [5]. PET provides functional information and allows metabolic measurements of malignant lesions. FDG PET has been successfully used for evaluation of tumor viability of a previously recognized active tumor after therapy. It can differentiate between scar tissue or active tumor tissue after treatment [6]. Many studies have shown the benefit of FDG PET in staging, restaging, and prediction of response to treatment in malignant lymphoma [7, 8]. The combination of metabolic and morphological imaging, using hybrid PET/CT scanners or software-based image fusion, is a promising technique that might overcome the limitations of each single modality [9–12]. By adding detailed anatomical information to the FDG PET, CT provides additional diagnostic information, thus enhancing the diagnostic accuracy in patients with malignant lymphoma.

The purpose of this study was to evaluate the accuracy of dedicated FDG PET, CT, and software-based image fusion. Both imaging modalities were performed for NHL or Hodgkin's disease. Results of this image fusion study may improve the quality of image interpretation in institutions, where both techniques are recorded at different locations.

## 2. Material and Methods

### 2.1. Patients

Seventy-seven patients with lymphoma treated at the Division of Haematology and Oncology, Medical University Innsbruck, Austria, were examined.

All patients underwent CT and PET examination followed by a software-based fusion of the two modalities. Thirteen patients underwent two PET/CT investigations, 8 patients had three PET/CT follow-up controls, and 1 patient obtained four PET/CT reinvestigations. Altogether 109 examinations of each modality were available. Among these, 14 scans were performed during initial staging, and 95 during restaging. Of the 77 included patients, 38 were female and 39 were male (age range 20–77, mean 53). All had histologically proven malignant lymphoma. Fifty-eight Patients suffered from NHL and 19 from HD. Of the 58 patients with NHL, 25 had diffuse large B cell lymphoma, 11 follicular lymphoma grade I-II, 6 follicular lymphoma grade III, 6 mantle cell lymphoma, 4 marginal zone lymphoma, 3 Burkitt's lymphoma, and 3 were unclassified according to the WHO classification [13, 14] (Table 1). Mean follow-up time was 14 months. In addition, all patients underwent conventional staging procedures, such as clinical examination, ultrasound, bone marrow biopsy, and laboratory screening.

### 2.2. CT Imaging Protocol

The CT examinations were obtained using a Siemens multislice CT scanner (Somatom Sensation Open, Erlangen, Germany). Patients were asked to empty their bladder before the scan in order to reduce imaging artifacts due to bladder filling. A bolus of Visipaque 320 (GE Healthcare, Chalfont St. Giles, UK), 2 mL per kg body weight, was injected intravenously. 3 mm slices of the neck, the thorax, and the abdomen were obtained in expiration.

### 2.3. PET Imaging Protocol

PET scans were performed on a GE Advance PET scanner (GE Medical Systems, Waukesha, WI, USA). All patients were required to fast for at least 8 hours prior to the PET scan in order to optimize the tumor's FDG uptake and to minimize the physiologic uptake in musculature, myocardium, and fat tissue. For this reason, a glucose level of 130 mg/dL, or below was mandatory. After intravenous injection of 370 MBq FDG, patients were prohibited to move and speak in order to minimize the physiologic muscle uptake. Though not routinely administered, oral benzodiazepines were available for nervous patients. Directly prior to imaging, patients were also asked to empty their bladders. The patients were placed in a comfortable supine position. The emission scan was started 60 minutes after the injection of the radioisotope. For attenuation correction, transmission scans were obtained by using a germanium 68 external line source. A whole-body acquisition was performed.

### 2.4. Reproducible Patient Fixation

A major problem in the acquisition and fusion of two different modalities is the repeatability of the patient's position between the two examinations. An exactly corresponding anatomical situation is required. Therefore, patients were positioned in a vacuum mattress (Bluebag, Medical Intelligence, Schwabmünchen, Germany) by an experienced technical assistant. The air is then withdrawn from the cushion, which turns the flexible cushion into a rigid fixation device allowing for exact repositioning of the patient. 4 external markers were subsequently attached to the mattress, positioned in the region of interest in every individual case. Image fusion was done separately by using external markers as reference, which are fixed to a vacuum mattress used to position the patient in exactly the same position in both scans.

### 2.5. Software-Based Fusion

For the image fusion, CT and PET studies were transferred to a Treon workstation (Medtronic Inc., Louisville, CO, USA) via intranet. Image registration can be done with external markers using the Cranial 4 multimodality software. With the CT data as a reference, 4 corresponding fiducials (markers) were identified in the two different datasets, and by using the paired-point matching algorithm the two datasets were then superimposed to each other.

### 2.6. Image Evaluation

An experienced nuclear medicine physician interpreted the 18-FDG PET images and an experienced radiologist read the CT images, blinded to the other imaging modality. The CT criteria for malignancy included the presence of organomegaly, an abnormal mass or structural changes inside a normal-sized organ. Lymph nodes were considered abnormal if the diameter was more than the standard CT size criteria for individual lymph node groups or because of abnormal contrast enhancement and central necrosis [15–17]. For FDG-PET, the criteria were the presence of abnormally increased tracer uptake at each suspected site. No quantification of FDG uptake of the lesions was done. All suspected findings at PET or at CT were documented. Afterwards, the findings of each method were discussed in an interdisciplinary image fusion conference. Finally, not only lymphoma lesions but also additional diagnostic findings from both modalities, like hernia, diverticulosis, or cholelithiasis, were documented.

To structure analysis of CT and PET images, the body was divided into 14 anatomic lymph node regions (cervical, nuchal, supraclavicular, infraclavicular, Waldeyer's ring, submandibular, axillary, hilar, mediastinal, pulmonary, upper abdominal, lower abdominal, iliac, and inguinal) and for each region, disease was recorded as present or absent. Thus, in total the large number of 1526, single regions were reviewed.

### 2.7. Reference Standards and Data Analysis

For the evaluation of tumor imaging methods, a major problem is the definition of the “gold standard” against which the different modalities should be compared. A histological confirmation of all sites suspicious of lymphoma involvement is virtually impossible. We defined the standard of reference as the sum of many factors including the clinical follow-up data (mean 14 months) such as physical examination or ultrasound, bone marrow and lymph node biopsy, and laboratory screening. Thus, the findings of each modality (PET, CT and software-based image fusion) were classified as true positive, true negative, false positive, or false negative. For the evaluation of the single CT and PET examination the results of the image fusion were also included to the “gold-standard.”

In order to analyse this study more precisely, we evaluated the results on a per patient analysis and on a lymph node basis. In the per patient analysis, any suspected lymph node in any of the defined regions leads to a positive result. If CT or PET findings were concordant to the “gold standard,” they were regarded as truly positive or truly negative. This is especially important due to the fact that most of the scans were performed during restaging (87%).

The lymph node region-based analysis should show whether a lymph node region is affected or not.

Finally, results of the fusion were compared with the clinical stage after complete followup. The patients were divided into subgroups (Hodgkin's disease, non-Hodgkin lymphoma, high-, and low-grade NHL).

### 2.8. Statistics

Statistical analysis was done utilizing the R system for statistical computation. Pearson's Chi-squared test with Yates' continuity correction and Fisher's exact test for count data were performed on all contingency tables by means of batch processing [18]. Sensitivity, specificity, positive predictive value, negative predictive value, and accuracy were calculated by standard methods.

Statistical analysis was performed using the Statistical Package for the Social Sciences for Windows (Release 13; SPSS Inc., Chicago, IL, USA).

## 3. Results

### 3.1. CT

In total, 162/1526 suspicious lymph node regions were detected by CT. The patient-based analysis (Table 2) showed an overall sensitivity and specificity of 90% and 76% according to 45 true positive and 45 true negative patients. The CT resulted in 5 false-negative and 14 false-positive patients. In patients with Hodgkin's disease a sensitivity of 93% and specificity of 62% were calculated, for patients with NHL 88% and 81%, respectively. For low-grade B-cell lymphoma, the sensitivity and specificity were 90% and 58% and for high-grade B-NHL 95% and 92%.

The lymph node region-based analysis revealed a sensitivity of 81% for detecting 133 true positive regions, 30 regions were false negative and 29 false positive (Table 3). By contrast, CT revealed also additional findings, for example, diverticulosis (*n* = 2), nephrolithiasis (*n* = 1), cholelithiasis (*n* = 2), aortic aneurysm (*n* = 1), abdominal-hernia (*n* = 3), a pancreatic cyst (*n* = 1), and a pericardial effusion (*n* = 1); which were not detected by FDG-PET.

### 3.2. PET

In total, FDG PET detected 172/1526 suspicious lymph node regions and showed a sensitivity of 94% and a specificity of 91% in the per patients analysis (Table 4). In 48 patients, the PET scan was true positive and in 53 patients true negative. 3 false-negative and 5 false-positive scans were noted. In the analysis of patients with Hodgkin's disease, the sensitivity and specificity were 93% and 87%. For all NHL subtypes it was 94% and 92%, respectively. In low-grade lymphomas, the sensitivity and specificity were 100% and 77%, in patients with high-grade lymphoma 91% and 96%.

Using the lymph node region-based evaluation, sensitivity of FDG-PET was 96% for all lymphomas (Table 5). With PET, 159 regions were true positive, 13 false positive, and only 6 false negative.

### 3.3. Image Fusion

Comparing the image fusion to the follow-up of the patients, 3 were found to be false positive and 106/109 were true positive which results in a sensitivity of 97%. In two patients, an axillary lymph node, which was suspected to be malignant in the image fusion, was identified as inflammatory by needle biopsy and histologic evaluation. A hypermetabolic mediastinal mass in one patient was not further investigated.

## 4. Discussion

To our knowledge, this is one of the largest cohort of patients with malignant lymphoma in which PET and CT scans are compared with each other.

The aim of this study was to evaluate if digitally fused CT and PET images can improve the diagnostic accuracy for the staging and monitoring of patients with malignant lymphoma. A substantially better result could be achieved by image fusion compared to CT or PET alone. In our study, image fusion was equivocal (false positive) in 3 cases out of 109 (2.8%), which is obviously more accurate than CT and PET alone. Thus, software-based fusion is an interesting alternative for hospitals that do not have access to an integrated PET/CT scanner. In addition, a retrospective image fusion of PET and CT, or even MRI images, allows most accurate image interpretation adopted to the individual patient. We used a paired-point matching algorithm to superimpose both datasets to each other, which differs from the automated rigid registration for image fusion as described earlier in the literature by Wolz et al. [19] and Slomka et al. [20]. To our experience in routine external fiducials are easier to detect in both datasets and allow for faster gained image fusion on navigation systems. Thus, image fusion using external fiducial markers is a simple and practical method which allows for routine use in clinical settings. The accuracy of image fusion depends largely on exact and stable positioning of the patient during scans, movement of internal organs, artefacts caused by PET attenuation correction, and misregistration of the two modalities.

Nevertheless, a limitation of our study is that no definition for a cut-off level for a “successful fusion” has been stated, and to date, no literature about the accuracy and success rate of the used fusion procedure exists.

Raanani et al. showed that additional diagnostic CT may be obviated in the PET/CT era, but not for all patients [21]. Osman et al. found a benefit for 3% of all cases when using a diagnostic scan—important findings may be overlooked without CT [22]. In our study, we found many important additional findings with CT, for example, diverticulosis, aortic aneurysm and nephrolithiasis; most of which cannot be seen with PET. Diagnostic CT is still an important tool and should not be relegated to being only a simple map for PET. The additional findings diagnosed by CT support the use of at least an initial diagnostic CT scan with intravenous contrast for the protocol of integrated PET/CT [23, 24].

**Figure 1:**
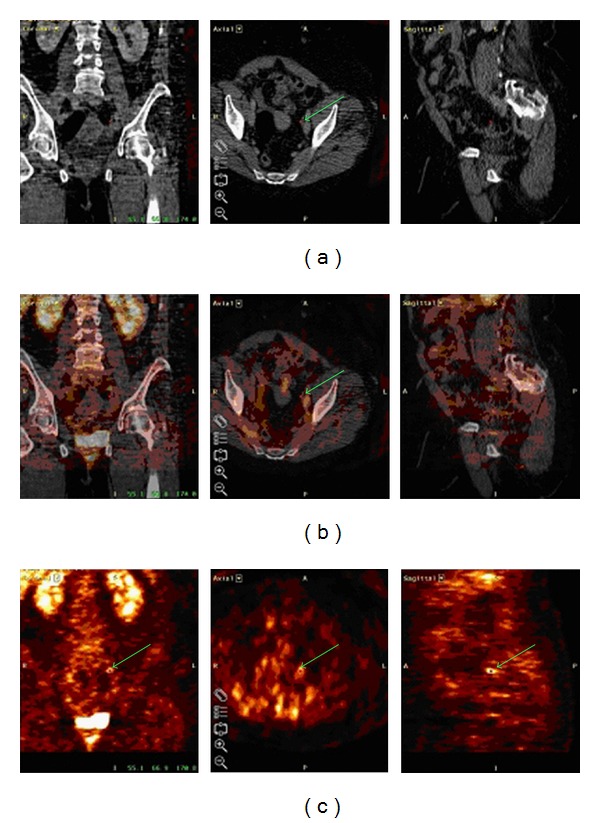
First row: computed tomography image; second row: software-based image fusion; third row: F18-FDG PET. Axial, coronal, and sagittal reformatted images of a patient with malignant lymphoma. F18-FDG PET (third row) shows a false-positive abdominal lesion (it was also scored as false positive) which turned out to be related to ureter-activity as showing by image fusion of PET with CT (second row). CT images did not show any pathologic enlarged lymph node. (first row).

In many studies, the advantage of PET in lymphoma [25] and its routine usage was suggested in the staging and follow-up of lymphoma patients [26, 27]. With FDG-PET our study reached a high sensitivity and specificity of 94% and 91% for all types of malignant lymphoma. These results are in concordance with the literature. Bangerter et al. showed a sensitivity of 96% and a specificity of 94% with PET in lymphoma of the thorax in 89 consecutive patients [8]. However, FDG is not a tumor-specific tracer. An increased glucose metabolism may also relate to other pathologic as well as physiologic conditions. In most of our cases it was possible to exclude false positive findings by the combination of CT and PET (Figure 1).

Surprisingly, the sensitivity and specificity for NHL were higher (94% and 92%) than in Hodgkin's disease, with 93% and 87%, respectively. An issue that is still under debate is the benefit of FDG PET in the staging and followup of low-grade lymphoma and among the different histologic subtypes. Some studies reported a higher sensitivity for high-grade than low-grade lymphoma [28–33]. Our study showed no significant difference between both, more precisely not significantly more false-negative and false-positive results in low-grade than in high-grade lymphoma—both could be detected by FDG PET. Our results are in concordance with studies that could also show no significant problems in detecting low-grade lymphoma [34–36]. However, the specificity in low-grade lymphoma in the patient-based statistic was only 77% compared to 96% in high-grade lymphoma. CT reached 92% sensitivity and only 44% specificity in low-grade lymphoma which is less accurate than PET. The study of Najjar et al. showed a sensitivity of 87% and a specificity of 87% with FDG PET in 36 patients with histological proven low-grade NHL, and CT had 90% and 100%, respectively [37].

In the lymph node region-based analysis, PET was true positive in 159 out of 165 lesions showing the high accuracy of FDG PET in detecting affected lymph nodes.

**Figure 2:**
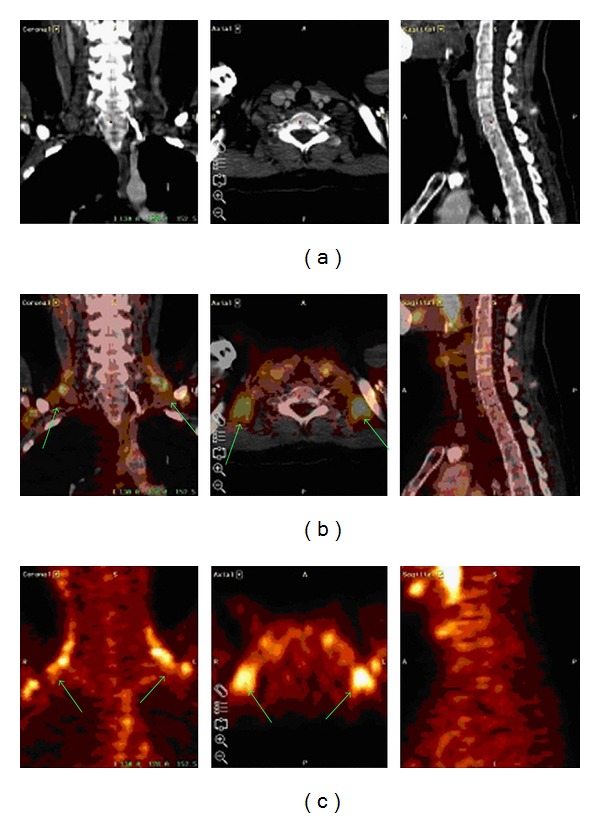
Axial, coronal, and sagittal reformatted images of a patient showing a typical “pitfall” with F18-FDG PET (third row). A positive bilateral cervical activity could be identified as activated brown fat tissue by using image fusion (second row). first row: computed tomography image; second row: software-based image fusion; third row: F18-FDG PET.

The low anatomical resolution of PET and the variability in normal physiologic FDG uptake in the body can be challenging, especially in the abdomen and pelvis [38] or cervical regions. However, such difficult anatomical locations and imaging pitfalls [39] can be circumvented by fusing CT and PET images (Figure 2). Similar to the data of La Fougère et al., an advantage of PET over CT alone was observed. Still, they could not show any significant difference between fused PET/CT and separate PET and CT image viewing side by side [40]. Another study also demonstrated the limitations of PET in the exact anatomical localisation of abdominal lesions [41].

The lymph node region-based analysis gives precise information about the accuracy in detecting affected lymph nodes by the methods applied. The results of this analysis show how many of the 14 defined regions were truly affected.

Due to the high number of true negative regions, the specificity of all the region-based analysis is not significant (Tables 3 and 5).

Freudenberg et al. had a sensitivity of 78% for CT and 86% for PET in restaging of 27 patients with lymphoma [42]. Weihrauch et al. showed a sensitivity of 74% and 88% for CT and PET but a specificity of 100% for both imaging modalities in staging Hodgkin's disease [43]. Our data, relating to sensitivity, are also compatible with the data of Stumpe et al., but their specificity of CT is only between 41% and 67% [44]. Schaefer et al. reported a sensitivity of 50% for contrast enhanced CT in detecting organic involvement with lymphoma. For statistical interpretation they defined only 6 lymph node regions and 5 organs for estimating organic involvement; in lymph node involvement, CT showed 88% sensitivity. They compared it to integrated PET/CT which showed 88% for organ involvement and 94% for lymph node involvement. They included 60 patients in their study [45]. Tatsumi et al. also used a combined PET/CT scanner to analyse lesions at the same anatomic locations in 53 patients with lymphoma. Their purpose was to compare FDG PET and CT in order to evaluate the frequency and causes of discrepant findings between both modalities. Of the 1537 determined anatomic sites, 48 showed discordant findings; 40 (83%) of these had correct PET findings, and only 5 had correct CT findings, 3 remained unresolved. However, in contrast to our study no intravenous contrast material was applied for the CT acquisition [46]. Our results with software-based image fusion in patients with malignant lymphoma correspond to results in surveys with integrated PET-CT scanners.

A limitation inherent to CT is that normal size or borderline lymph nodes harbouring a malignant and active disease may be diagnosed as normal because no pathologic criteria as size, shape or contrast enhancement can be identified. There is also a problem in differentiation between malignant and inflammatory enlargement of lymph nodes. However, our study with CT showed an excellent sensitivity of 90% in detecting a malignant process (per patient) and 81% for detecting malignant lymph node regions. One reason for the better results compared to other studies might be the technical benefit of the multislice CT scanner and the application of contrast media. The 14 false-positive patients were due to enlarged lymph nodes that had not yet shrunk after the therapy or because of benign “reactive” lymph nodes. This is explained by the fact that the decrease of lymph node size does not follow a decrease of the metabolic activity.

Remarkably, the specificity of CT in Hodgkin's disease and low-grade NHL were only 62% and 44% in the patient-based evaluation—we could find no possible explanation for that.

The poorest sensitivity for CT (79%) in our study was achieved in HD with a total of 14 false-positive and 14 false-negative lymph node regions.

FDG PET was responsible for 13 false-positive lymph node regions which caused 5 false-positive patients. Without combining PET with an anatomical method, this could result in crucial wrong decisions in treatment management.

## 5. Conclusion

The aim of this study was to evaluate the clinical significance of CT and PET alone compared to a combination by using software-based fusion. Image fusion is a helpful technique for both staging and followup in patients with malignant lymphoma. Differences between PET and CT sensitivities were not significant but the combination of both technologies is considerably more accurate than each modality on its own, posing an interesting alternative for hospitals that do not have access to an integrated PET/CT scanner.

## Figures and Tables

**Table 1:** Histological NHL subtypes.

Pathological NHL subtypes	Number of patients
Diffuse large B cell lymphoma	25
Follicular lymphoma grade I-II	11
Follicular lymphoma grade III	6
Mantle cell lymphoma	6
Marginal zone lymphoma	4
Burkitt lymphoma	3
Unclassified	3

**Table 2:** Results of patient-based analysis with CT.

	TP	TN	FP	FN	*N*	sens.	spec.	PPV	NPV	acc.	*χ*′′	*P*(*χ*²)	*P*(*F*)
Malignant lymphoma	45	45	14	5	109	90	76	76	90	82	45.2	<0.0001	<0.0001
Morbus Hodgkin	14	10	6	1	31	93	62	70	90	77	8.2	0.0041	0.0021
NHL	31	35	8	4	78	88	81	79	89	84	35.0	<0.0001	<0.0001
Low-grade NHL	9	7	5	1	22	90	58	64	87	72	3.6	0.057	0.031
High-grade NHL	22	25	2	1	50	95	92	91	96	94	35.3	<0.0001	<0.0001

TP: true positive, TN: true negative, FP: false positive, FN: false negative, *N*: number of subjects, sens.: sensitivity, spec.: specificity, PPV: positive predictive value, NPV: negative predictive value, acc.: accuracy, *P*(*χ*²): *P* value for Pearson's Chi-squared test with Yates' continuity correction, and *P*(*F*): *P* value for Fisher's Exact Test for Count Data.

**Table 3:** Results of lymph node region-based analysis with CT.

	TP	TN	FP	FN	*N*	sens.	spec.	PPV	NPV	acc.	*χ*′′	*P*(*χ*²)	*P*(*F*)
Malignant lymphoma	133	1334	29	30	1526	81	97	82	97	96	961	<0.0001	<0.0001
Morbus Hodgkin	53	353	14	14	434	79	96	79	96	93	240	<0.0001	<0.0001
NHL	80	981	15	16	1092	83	98	84	98	97	728	<0.0001	<0.0001
Low-grade NHL	20	278	5	5	308	80	98	80	98	96	178	<0.0001	<0.0001
High-grade NHL	60	624	7	9	700	86	98	89	98	97	520	<0.0001	<0.0001

Column headings as in Table 2, with *N*: number of regions.

**Table 4:** Results of patient-based analysis with PET.

	TP	TN	FP	FN	*N*	sens.	spec.	PPV	NPV	acc.	*χ*′′	*P*(*χ*²)	*P*(*F*)
Malignant Lymphoma	48	53	5	3	109	94	91	90	94	92	76.0	<0.0001	<0.0001
Morbus Hodgkin	14	14	2	1	31	93	87	87	93	90	17.2	<0.0001	<0.0001
NHL	34	39	3	2	78	94	92	91	95	93	55.8	<0.0001	<0.0001
Low-grade NHL	13	7	2	0	22	100	77	86	100	90	11.5	0.0007	0.0002
High-grade NHL	21	26	1	2	50	91	96	95	92	94	35.2	<0.0001	<0.0001

Column headings as in Table 2.

**Table 5:** Results of lymph node region-based analysis with PET.

	TP	TN	FP	FN	*N*	sens.	spec.	PPV	NPV	acc.	*χ*′′	*P*(*χ*²)	*P*(*F*)
Malignant lymphoma	159	1348	13	6	1526	96	99	92	99	98	1330	<0.0001	<0.0001
Morbus Hodgkin	66	361	6	1	434	98	98	91	99	98	377	<0.0001	<0.0001
NHL	93	987	7	5	1092	94	99	93	99	98	940	<0.0001	<0.0001
Low-grade NHL	23	280	3	2	308	92	98	88	99	98	234	<0.0001	<0.0001
High-grade NHL	66	627	4	3	700	95	99	94	99	99	613	<0.0001	<0.0001

Column headings as in Table 2, with *N*: number of regions.
